# Expression profiling and bioinformatics analysis of serum exosomal circular RNAs in lymph node metastasis of papillary thyroid carcinoma

**DOI:** 10.7555/JBR.37.20230304

**Published:** 2024-05-30

**Authors:** Huiyong Peng, Zhangwei Zhu, Jie Xing, Qian Xu, Changfeng Man, Shengjun Wang, Yingzhao Liu, Zhengdong Zhang

**Affiliations:** 1 Department of Environmental Genomics, Jiangsu Key Laboratory of Cancer Biomarkers, Prevention and Treatment, Collaborative Innovation Center for Cancer Personalized Medicine, School of Public Health, Nanjing Medical University, Nanjing, Jiangsu 211166, China; 2 Department of Genetic Toxicology, the Key Laboratory of Modern Toxicology of Ministry of Education, Center for Global Health, School of Public Health, Nanjing Medical University, Nanjing, Jiangsu 211166, China; 3 Department of Laboratory Medicine, Zhenjiang Medical School of Nanjing Medical University, Zhenjiang, Jiangsu 212002, China; 4 Department of Endocrinology, Zhenjiang Medical School of Nanjing Medical University, Zhenjiang, Jiangsu 212002, China; 5 Department of Oncology, Zhenjiang Medical School of Nanjing Medical University, Zhenjiang, Jiangsu 212002, China; 6 Department of Laboratory Medicine, the Affiliated Hospital of Jiangsu University, Zhenjiang, Jiangsu 212008, China

**Keywords:** papillary thyroid carcinoma, exosome, circular RNA, regulatory network, lymph node metastasis

## Abstract

Most papillary thyroid carcinoma (PTC) patients have a good prognosis. However, lymph node metastasis (LNM), the most common manifestation of disease progression, is frequently associated with a poor prognosis. Nevertheless, few studies have focused on the underlying mechanisms of LNM. In the current study, we aimed to investigate the potential role of exosomal circRNAs that contribute to LNM in PTC. We identified 9000 differentially expressed exosomal circRNAs in PTC patients with LNM, including 684 upregulated and 2193 downregulated circRNAs. Functional enrichment analysis revealed that these differentially expressed circRNAs were primarily involved in a variety of molecular and signaling pathways correlated with PTC progression and LNM. Through bioinformatics analysis, we identified 14 circRNA-miRNA-mRNA networks related to LNM-associated signaling pathways in PTC. Moreover, both *circTACC2*-miR-7-*EGFR* and *circBIRC6*-miR-24-3p-*BCL2L11* axes were verified for their potential involvement in PTC with LNM. Additionally, we identified four upregulated circRNA-related hub genes and eight hub genes correlated with downregulated circRNAs, some of which were validated as being potentially involved in LNM in PTC. Collectively, our findings provide a novel framework for an in-depth investigation of the function of dysregulated exosomal circRNAs and their potential as biomarkers in PTC patients with LNM.

## Introduction

Thyroid cancer is a common primary endocrine tumor, accounting for approximately 2% of all malignancies in the body^[1]^. Among its subtypes, papillary thyroid carcinoma (PTC) is the most frequent subtype, characterized by goiter, nodules, a hard and fixed texture, an irregular shape, and an unclear boundary^[2–3]^. PTC affects millions of people worldwide, with an annual incidence of 4.6–14.4 cases per 100000 individuals globally^[1]^, and it is more common in females and high-iodine countries^[4]^. Although the mortality rate among patients with PTC has remained relatively low and stable over the last few decades because of surgical procedures and radioactive iodine treatment, and although PTC is often referred to as an indolent neoplasm, some patients still die from advanced PTC^[5]^. Among the progressive stages of PTC, lymph node metastasis (LNM) is the most prominent manifestation, and it is a key indicator for assessing the prognosis and surgical options for PTC patients^[6]^. To develop effective diagnostic and therapeutic approaches, more innovative targets must be identified to explore the underlying mechanisms of LNM in PTC.

Exosomes, as biological signal transmitters and carriers, act as messengers for intercellular communication in the extracellular microenvironment. Exosomes are natural, phospholipid-based bilayer membrane vesicles synthesized and released by cells, with a diameter ranging from 30 to 150 nm^[7]^. Extensive evidence has revealed that exosomes are usually present in various body fluids and play a pivotal role in tumor biology. Exosomes from secretory cells may transport biological components to recipient cells within the tumor microenvironment (TME), thereby participating in tumor development^[8]^. Luan *et al*^[9]^ demonstrated that exosomes derived from melanoma cells could transport miR-106b-5p to melanocytes, participating in the epithelial-mesenchymal transition. Moreover, Thakur *et al*^[10]^ demonstrated that exosomal DNA could be used as a potential circulating biomarker to identify tumor-associated genetic mutations. As such, exosomes may potentially serve as novel biomarkers for the diagnosis and treatment of tumors.

Circular RNAs (circRNAs), which are among the important molecules in exosomes, have attracted the interest of investigators in recent years. Accumulating evidence has indicated that circRNAs are significantly correlated with the development of multiple cancers^[11–12]^. Unlike other RNAs, circRNAs have a covalently closed cyclic structure, which makes them resistant to degradation by ribonuclease R within the organism^[13]^. Moreover, circRNAs exhibit cell-type-specific expression and perform different functions based on their intracellular localization^[14]^. Although investigators have focused on circRNAs and their roles in the progression of PTC^[15]^, the majority of circRNA studies have been confined to cancer cells. The role of exosomal circRNAs in LNM among PTC patients remains unclear.

In the current study, we aimed to investigate the role of exosomal circRNAs in PTC patients with LNM. We first identified differentially expressed circRNAs derived from serum exosomes. Then, we determined the potential roles of significantly dysregulated circRNAs through bioinformatic analysis and preliminary verification.

## Materials and methods

### Subjects and specimens

Five PTC patients with LNM, aged 28 to 67 years, were enrolled from the Zhenjiang Clinical Medical School of Nanjing Medical University. The cases were diagnosed based on clinical manifestations and B-mode ultrasonography, and the diagnosis was then confirmed by pathologists after surgery. Five sex- and age-matched healthy adult subjects were included as healthy controls. Subjects with tumors, thyroid diseases, autoimmune diseases, or chronic infectious diseases were excluded. The peripheral blood parameters of all cases were within the normal range. An additional 14 PTC patients with LNM and 13 healthy controls were included as validation samples. There were no significant differences in age (*P* = 0.566) or sex (*P* = 0.586) between cases and controls. Peripheral blood samples (5 mL) were collected from each subject. After blood coagulation, the serum was obtained through centrifugation at 500 *g* for 20 min. The isolated serum samples were stored at −80 ℃.

The study was approved by the Institutional Review Board of the First People's Hospital of Zhenjiang (Approval No. K-20200012-Y), and was carried out in accordance with the Helsinki Declaration. Informed consent to access medical records and publish clinical data was obtained from each participant.

### Cell culture

Human thyroid normal cell line (Nthy-ori 3-1) and PTC metastatic cell line (BCPAP) were cultured in RPMI-1640 medium (Gibco, Waltham, MA, USA) supplemented with 10% fetal bovine serum (Gibco) at 37 ℃ in a 5% CO_2_. These cell lines were used for validation of exosomal circRNAs and potential regulatory genes by quantitative reverse transcription-PCR (qRT-PCR) analysis.

### Exosome isolation and identification

The serum samples were centrifuged at 3000 *g* at 4 ℃ for 10 min to remove cells and cell debris. The supernatant after centrifugation was then filtered through a 0.22-µm filter to remove larger particulate matter. Subsequently, exosomes were extracted using the QIAGEN exoEasy Maxi Kit (Cat. #76064, QIAGEN, Dusseldorf, NRW, Germany) according to the manufacturer's instructions. The following methods were used to identify exosomes: (1) Transmission electron microscopy with the Tecnai G2 (FEI, Hillsboro, TX, USA) was performed to determine the shape and size of exosomes. (2) Nano-flow cytometry with the NanoFCM U30 (NanoFCM, Inc., Xiamen, China) was performed to examine the size distribution of exosomes. (3) The protein markers of exosomes were determined by Western blotting analysis. Antibodies against positive markers, *i.e.*, CD63 (1∶2000, Cat. #ab216130, Abcam, Cambridge, UK) and CD81 (1∶2000, Cat. #ab109201, Abcam), and negative marker calnexin (1∶5000, Cat. #ab22595, Abcam) were used.

### Library preparation of RNA and circRNA sequencing

Total RNA was obtained from serum exosomes using the exoRNeasy Midi Kit (Cat. #77144, QIAGEN). A NanoDrop ND-1000 spectrophotometer was used to detect the concentration and quality of RNA (Thermo Fisher Scientific, Waltham, MA, USA). Next-generation sequencing (NGS) was performed by Cloud-Seq Biotech, Inc. (Shanghai, China). After the removal of ribosomal RNA, the sequencing libraries were constructed using the GenSeq^®^ Low Input RNA Library Prep Kit (Cloud-Seq Biotech). The constructed sequencing libraries were quality controlled and quantified by Q30 with the BioAnalyzer 2100 system (Agilent, Santa Clara, CA, USA), followed by 150 bp double-ended sequencing using the Illumina NovaSeq 6000 (Illumina, Inc., San Diego, CA, USA).

### circRNA profiling analysis

Cutadapt software (v1.9.3) was used to obtain high-quality reads. These reads were then compared to the reference genome/transcriptome using STAR software (v2.5.1b), and circRNAs were identified using DCC software (v0.4.4). Subsequently, the identified circRNAs were annotated using the circBase database (http://www.circbase.org/). The data were standardized, and the differentially expressed circRNAs were screened using edgeR software (v3.16.5) and were presented as logarithmic counts per million. circRNAs with a fold change ≥ 2.0 and a *P*-value < 0.05 were determined to be significantly dysregulated.

### Functional analysis of identified circRNAs

The Gene Ontology (GO; http://www.geneontology.org) enrichment analysis of host genes was performed to annotate and speculate on the functions of their corresponding circRNAs, including biological process (BP), cellular component (CC), and molecular function (MF). Additionally, the potential enrichment signaling pathways of circRNAs were predicted by the Kyoto Encyclopedia of Genes and Genomes (KEGG) (https://www.genome.jp/kegg/) analysis of circRNA-derived host genes.

### Construction of the competitive endogenous RNA (ceRNA) network

The ceRNA regulatory network is an important regulatory mode among circRNA, miRNA, and mRNA. The potential sponging relationships between circRNAs and miRNAs were predicted using miRanda (v3.3) and TargetScan software (v8.0). The intersection of the results from four prediction programs, including miRWalk, miRDB, miRTarBase, and TargetScan, was obtained to predict the target genes of miRNAs. Cytoscape software (v3.8.2) was used to construct the circRNA-miRNA-mRNA regulatory networks. The functions of mRNAs in regulatory networks were further determined through the GO and KEGG pathway enrichment analyses. Furthermore, the predicted miRNAs and genes were screened through the existing studies to identify which miRNAs had been reported to show an inverse expression trend to their corresponding circRNAs in PTC and which genes had been documented to be correlated with PTC patients with LNM.

### qRT-PCR analysis

Total RNA was extracted from cell lines using an RNA rapid extraction kit (Yishan, Shanghai, China) according to the manufacturer's instructions. cDNA reverse transcription was carried out using the ReverTraAca^®^ RT-qPCR kit (Toyobo, Osaka, Japan) according to the manufacturer's instructions. TB Green^®^ Premix Ex Taq Ⅱ (Takara, Osaka, Japan) was used to amplify cDNA in the ABI7500 instrument (Applied Biosystems, Foster City, CA, USA). The primers are summarized in ***Supplementary Table 1*** (available online). The transcript levels of circRNAs and mRNAs were normalized to actin beta (*ACTB*).

### Integration of protein-protein interaction (PPI) network

The STRING database (https://string-db.org/) was used to predict the potential relationships between circRNAs and protein-coding genes, and PPI networks were generated to describe the interactions among these protein-coding genes using Cytoscape software (PPI score > 0.9). CentiScaPe, a Cytoscape plugin, was used to calculate the degree centrality. The Molecular Complex Detection algorithm (MCODE) was used to determine the hub modules in the constructed PPI networks. Subsequently, the hub genes were identified based on degree centrality and module clustering^[16]^.

### Expression analysis by using the database

The transcript levels of potential regulatory genes were analyzed in the TCGA-THCA database (https://portal.gdc.cancer.gov/projects/TCGA-THCA). The database contained the sequencing data of 24 PTC patients with LNM and paired normal thyroid tissues. Additionally, immunohistochemical data for the potential regulatory genes in PTC and normal thyroid tissues were downloaded from the Human Protein Atlas (HPA) database (https://www.proteinatlas.org/). The staining intensity was classified into four categories: not detected, low, medium, and high.

### Statistical analysis

The sequencing data were analyzed using R software. Quantitative data were presented as mean ± standard deviation, and significant differences between two groups were assessed using Student's *t*-test or *χ*^*2*^ test (SPSS 16.0.0.247 software). A *P*-value < 0.05 was considered statistically significant.

## Results

### Identification of exosomes in the serum

**Figure 1:**
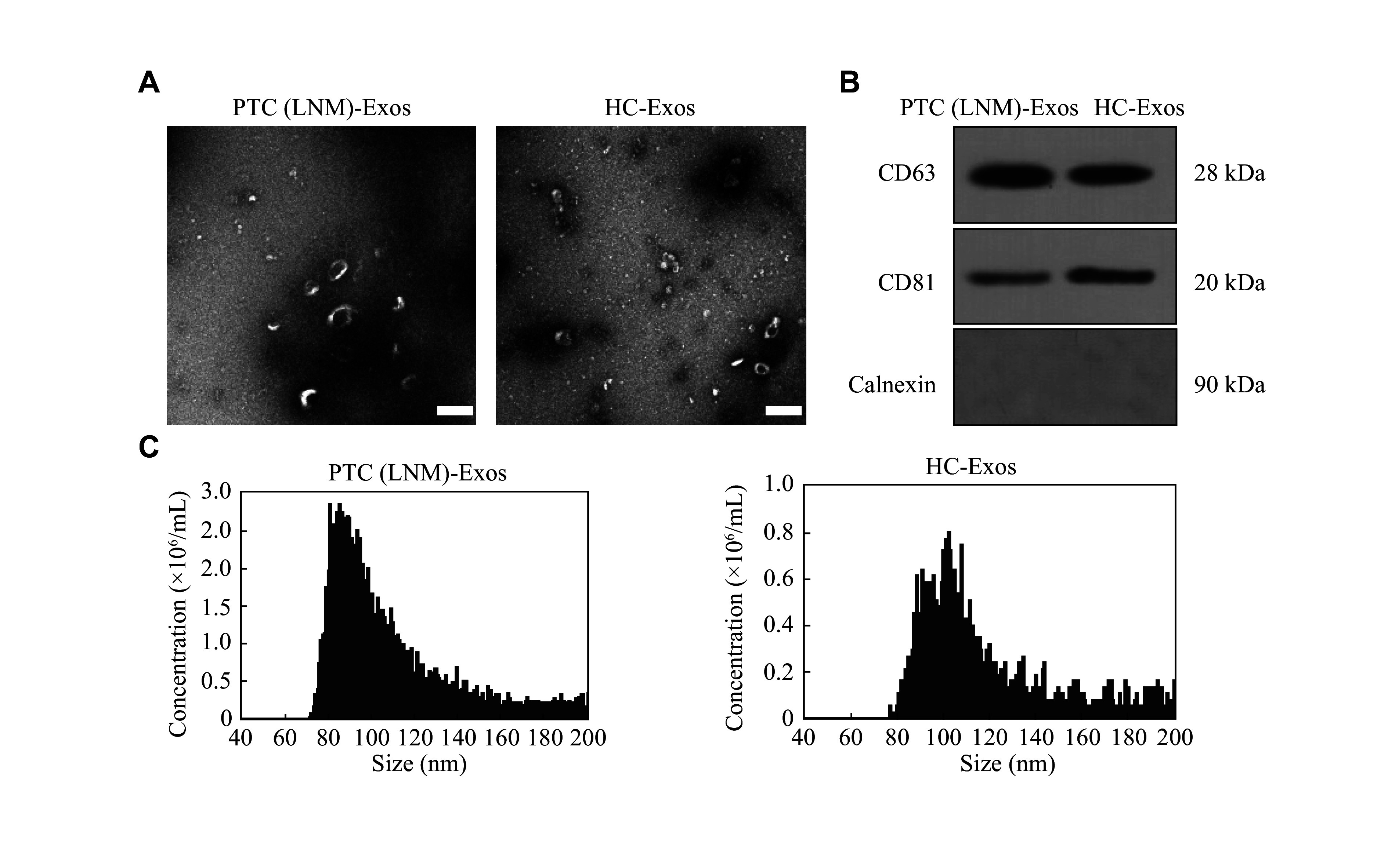
Identification of serum exosomes. A: Representative images of exosomes derived from PTC patients with LNM [PTC (LNM)-Exos] and healthy controls (HC-Exos). The morphology of exosomes was determined by transmission electron microscopy. Scale bar, 100 nm. B: The markers of exosomes were evaluated by Western blotting analysis. CD63 and CD81 were used as positive markers, and calnexin as a negative marker. C: The size distribution of serum exosomes was determined by NanoFCM analysis. Abbreviations: PTC, papillary thyroid carcinoma; LNM, lymph node metastasis.

We collected peripheral blood from PTC patients with LNM and healthy controls to extract exosomes from human serum, and designated the exosomes as PTC (LNM)-Exos and HC-Exos, respectively. Characterization of the exosomes showed that PTC (LNM)-Exos and HC-Exos exhibited a typical cup-shaped morphology (***Fig. 1A***). Additionally, serum exosomes from both groups expressed the exosomal markers (CD63 and CD81) but were negative for calnexin, a cellular constituent used as a negative protein marker of exosomes (***Fig. 1B***). Subsequently, we analyzed the size of exosomes using the NanoFCM analysis and found that the size of exosomes was approximately 80–100 nm in diameter (***Fig. 1C***). These results indicated that the extracted serum components were exosomes, providing a basis for further experiments.

### Expression profiling analysis of serum exosomal circRNAs

**Figure 2:**
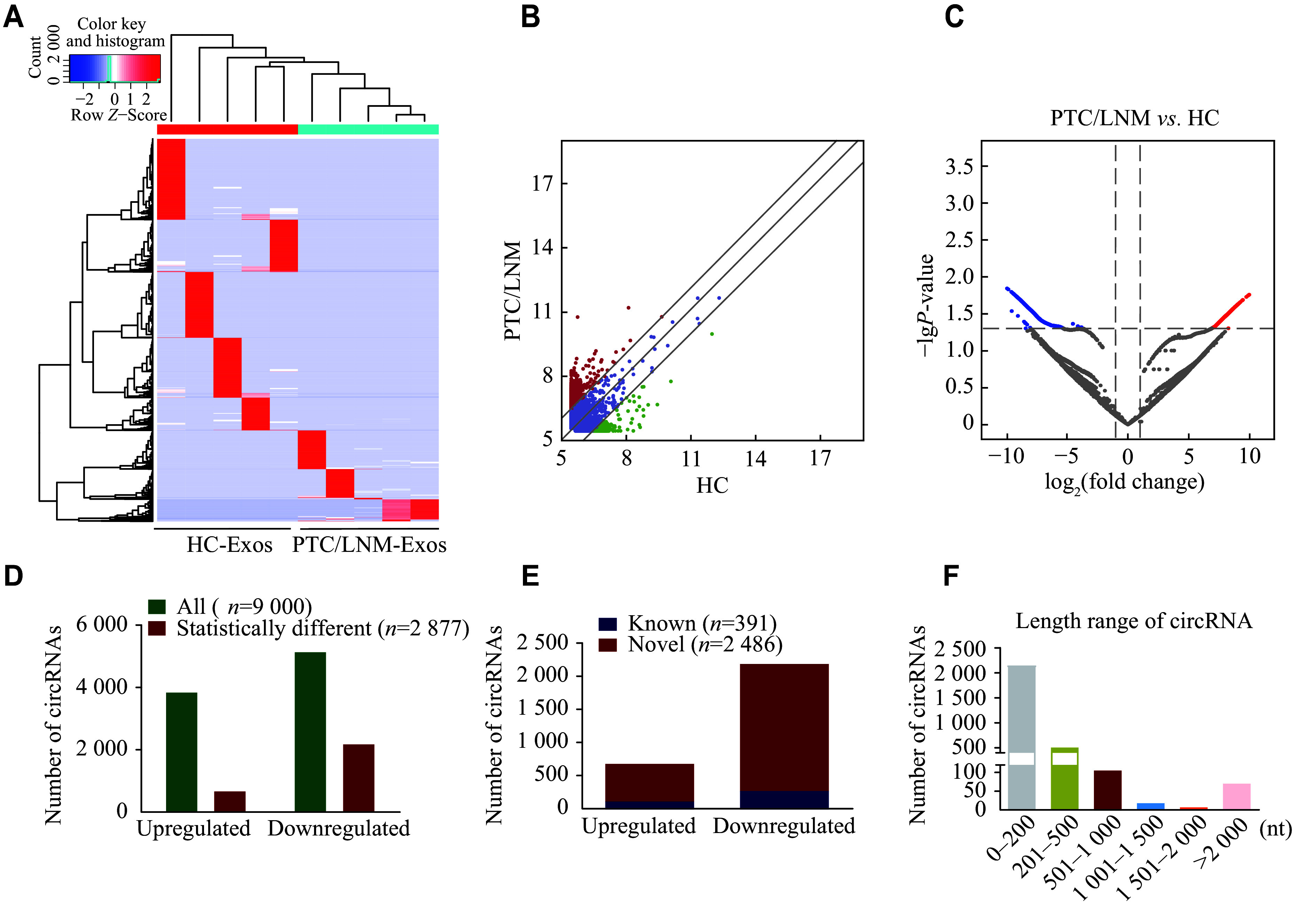
Expression profiling of serum exosomal circRNAs in PTC patients with LNM. Serum exosomes derived from five PTC patients with LNM (PTC/LNM) and five healthy controls (HC) were subjected to NGS analysis. A: Hierarchical clustering analysis of dysregulated circRNAs in serum exosomes. B and C: Scatter plot and volcano plot illustrating the significantly dysregulated circRNAs. Red (upregulated) and green/blue (downregulated) dots indicate exosomal circRNAs with more than a 2-fold change between the two groups. D: The statistical diagram of dysregulated circRNAs in serum exosomes between PTC patients with LNM and healthy controls. E: The statistical diagram of novel circRNAs (red) and previously identified circRNAs (blue). F: The length distribution of 2877 significantly dysregulated circRNAs. Abbreviations: PTC, papillary thyroid carcinoma; LNM, lymph node metastasis.

The expression profile of exosome circRNAs was examined using NGS (GEO ID: GSE247120). Hierarchical cluster analysis revealed distinct patterns of circRNAs between the PTC (LNM)-Exos and HC-Exos groups (***Fig. 2A***). There were 9000 dysregulated circRNAs between the two groups, including 3852 upregulated and 5148 downregulated circRNAs. Scatter plots (***Fig. 2B***) and volcano plots (***Fig. 2C***) were used to identify significantly dysregulated circRNAs, among which 2877 circRNAs, including 684 upregulated and 2193 downregulated circRNAs, showed significant differences (***Fig. 2D***). Intriguingly, the majority of these were novel circRNAs, with 2486 circRNAs (569 upregulated and 1917 downregulated), while the rest were known circRNAs, including 115 upregulated and 276 downregulated circRNAs (***Fig. 2E***). These circRNAs ranged in length from 116 to 81282 nt, with the majority in the group of 0–200 nt, accounting for 75.04% (***Fig. 2F***).

**Figure 3:**
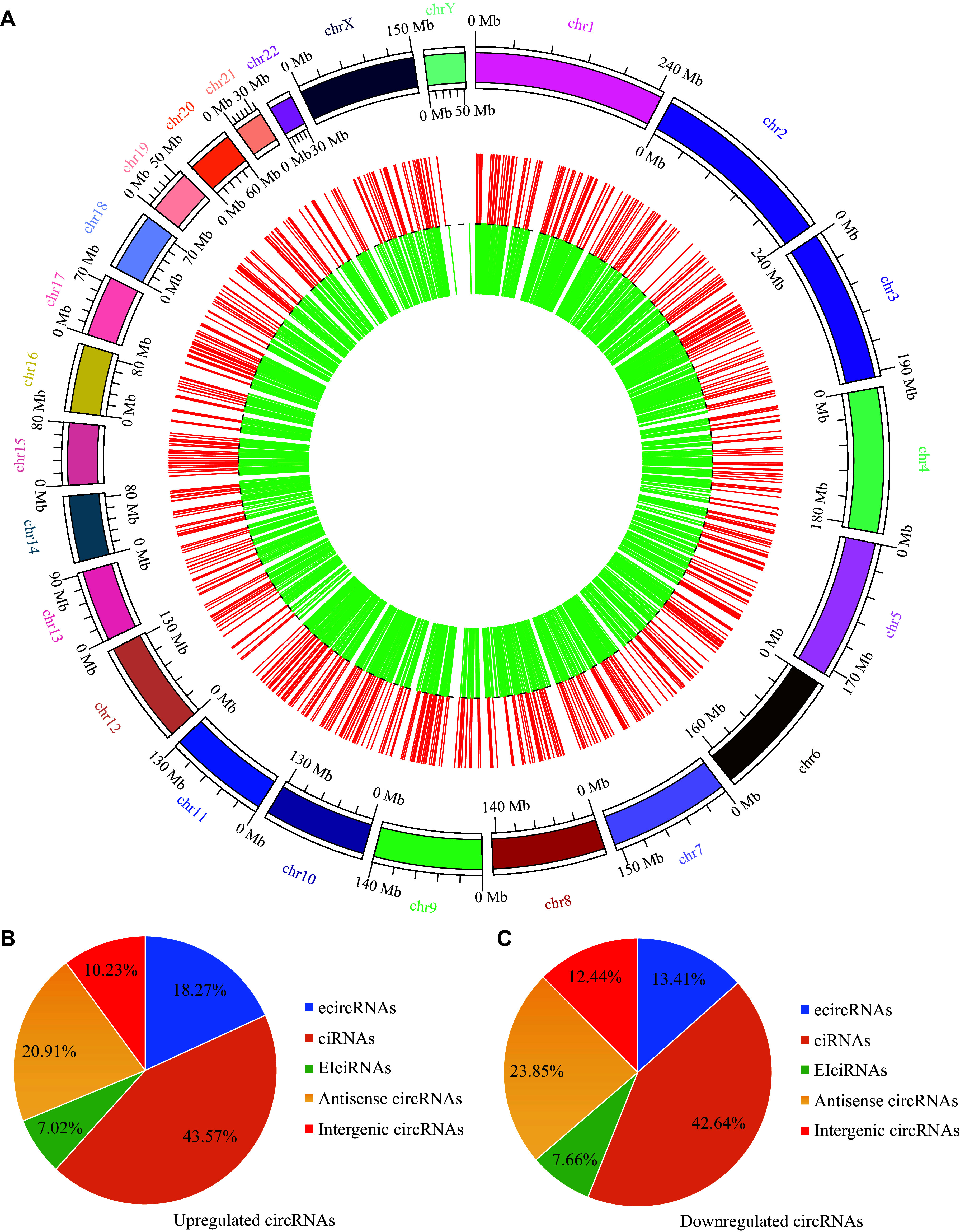
Classification of significantly dysregulated circRNAs. A: The distribution of significantly dysregulated circRNAs on human chromosomes. B and C: The categories of significantly upregulated (B) and downregulated (C) circRNAs. Abbreviations: ecircRNAs, exonic circRNAs; ciRNAs, intronic circRNAs; EIciRNAs, exon-intron circRNAs.

Next, we analyzed the human chromosomal distribution of the 2877 circRNAs and found that these significantly dysregulated circRNAs were distributed across all chromosomes, with the majority concentrated on chromosome 1 (***Fig. 3A***). Additionally, circRNAs were classified into five categories according to the source of circRNA sequences. Among them, circular intronic RNAs accounted for the largest proportion, while exon-intron circRNAs accounted for the smallest proportion in both upregulated (***Fig. 3B***) and downregulated circRNAs (***Fig. 3C***). Together, these data indicated that the differentially expressed circRNAs were present in exosomes derived from PTC patients with LNM and healthy controls.

### Bioinformatics analysis of the notably dysregulated circRNAs

**Figure 4:**
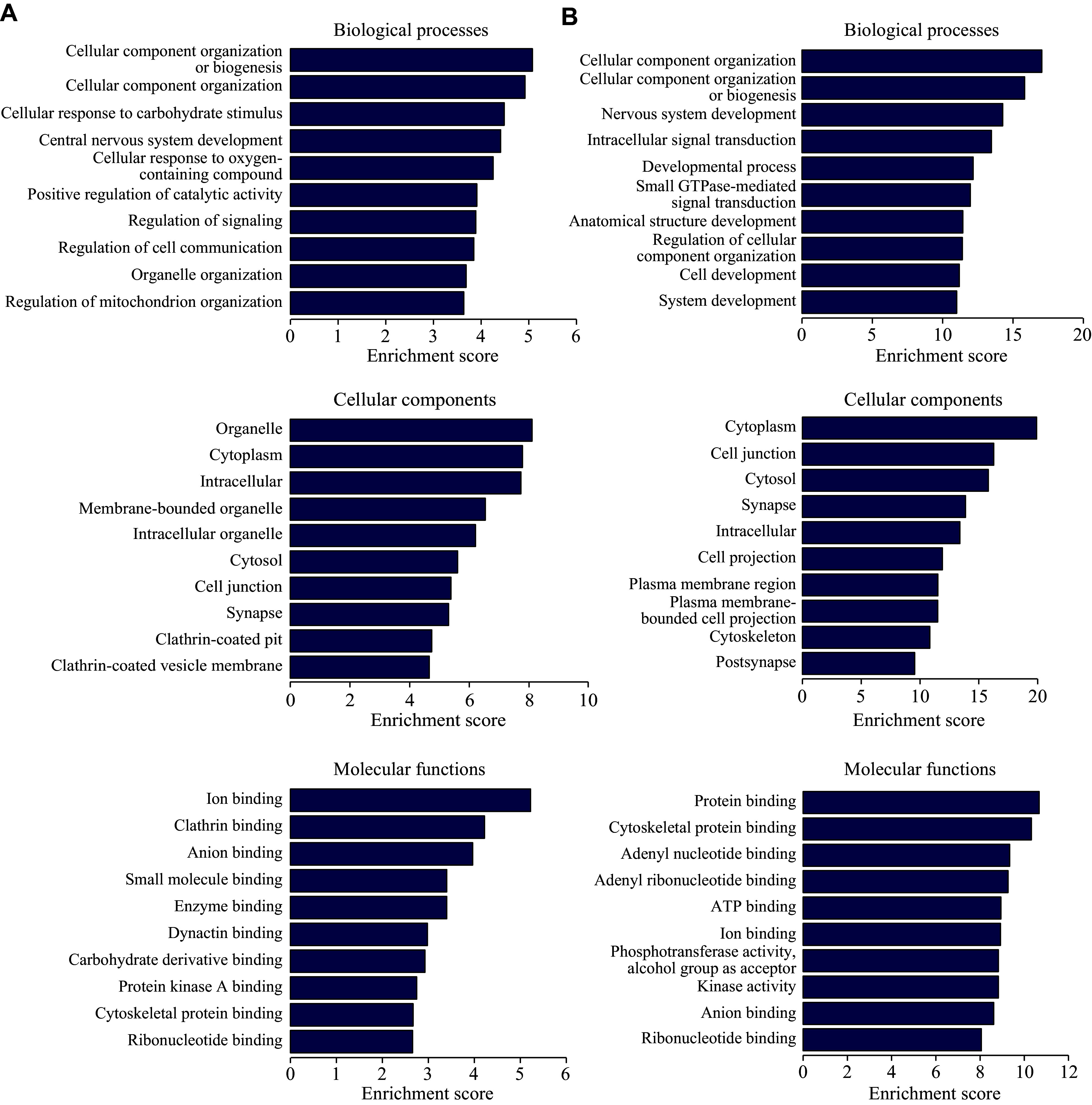
GO enrichment analysis of significantly dysregulated circRNAs. GO terms encompass three categories, including biological process, cellular component, and molecular function. A and B: The top 10 GO terms correlated with upregulated (A) and downregulated (B) circRNAs.

The potential biological functions of differentially expressed circRNAs were determined by GO enrichment analysis. A total of 679 GO terms associated with upregulated circRNAs were identified, and the top 10 GO terms are presented in ***Fig. 4A***. For downregulated circRNAs, 1435 GO enriched terms were identified as statistically significant, and the top 10 GO terms are shown in ***Fig. 4B***. In terms of BP, these circRNAs were closely related to cell components, cell communication, and a series of signaling transduction processes. The correlation between these circRNAs and the CC terms was mainly concentrated in the cytoplasm, organelle, synapse, clathrin, and vesicle. Regarding the MF terms, these circRNAs exhibited a strong capability to bind various proteins, enzymes, and nucleotides.

**Figure 5:**
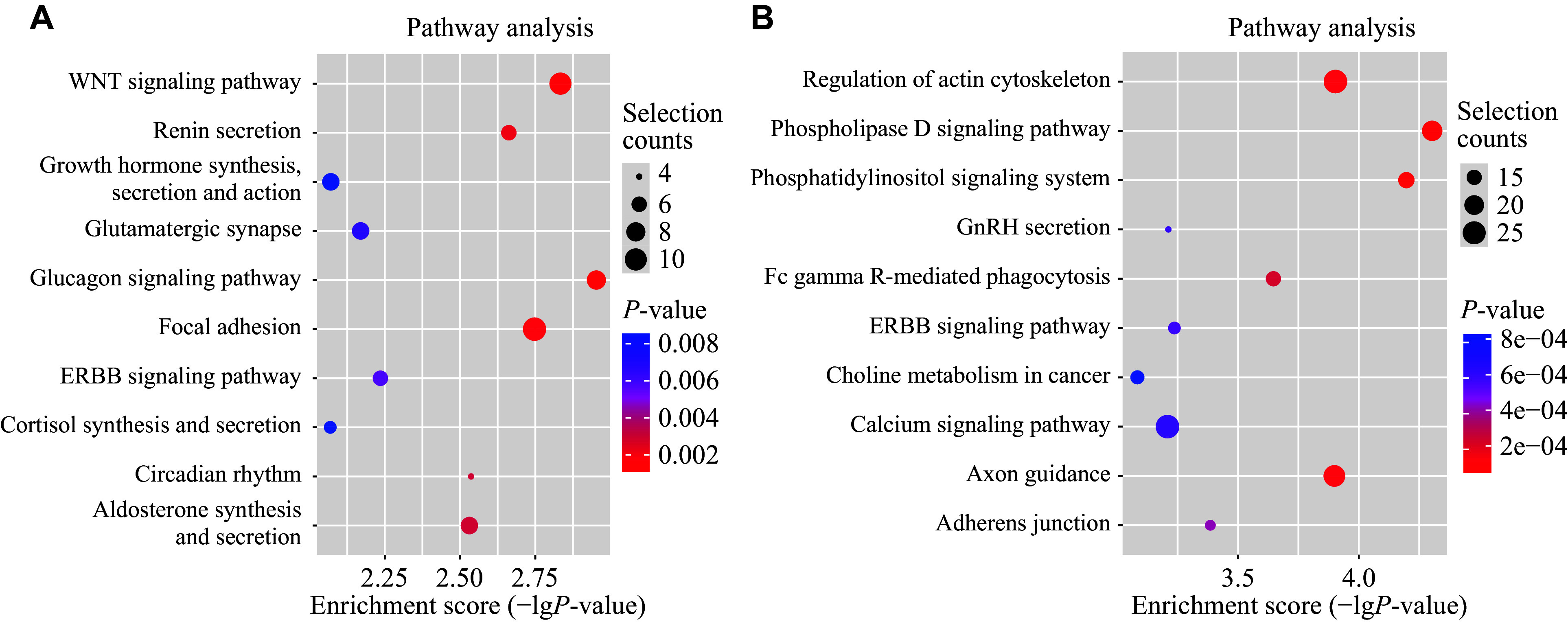
KEGG pathway enrichment analysis of significantly dysregulated circRNAs. Molecular datasets from genomics, transcriptomics, proteomics, and metabolomics were mapped onto KEGG pathways to infer the correlated pathways of these molecules. A and B: The top 10 KEGG pathway terms correlated with upregulated (A) and downregulated (B) circRNAs.

The potential signaling pathways correlated with dysregulated circRNAs were determined by the KEGG pathway analysis of their host genes. According to the KEGG classification, a total of 91 significantly enriched signaling pathways were identified, including 34 pathways correlated with upregulated circRNAs and 57 pathways involved in downregulated circRNAs. Among them, 15 signaling pathways were closely related to both upregulated and downregulated circRNAs. The top 10 enriched signaling pathways for upregulated and downregulated circRNAs are shown in ***Fig. 5A*** and ***5B***, respectively. The WNT, ERBB, and calcium signaling pathways have been reported as key factors in the progression of PTC^[17–19]^. These findings suggest that dysregulated exosomal circRNAs may play a significant role in PTC with LNM.

### Interactions among circRNAs, miRNAs, and mRNAs

**Figure 6:**
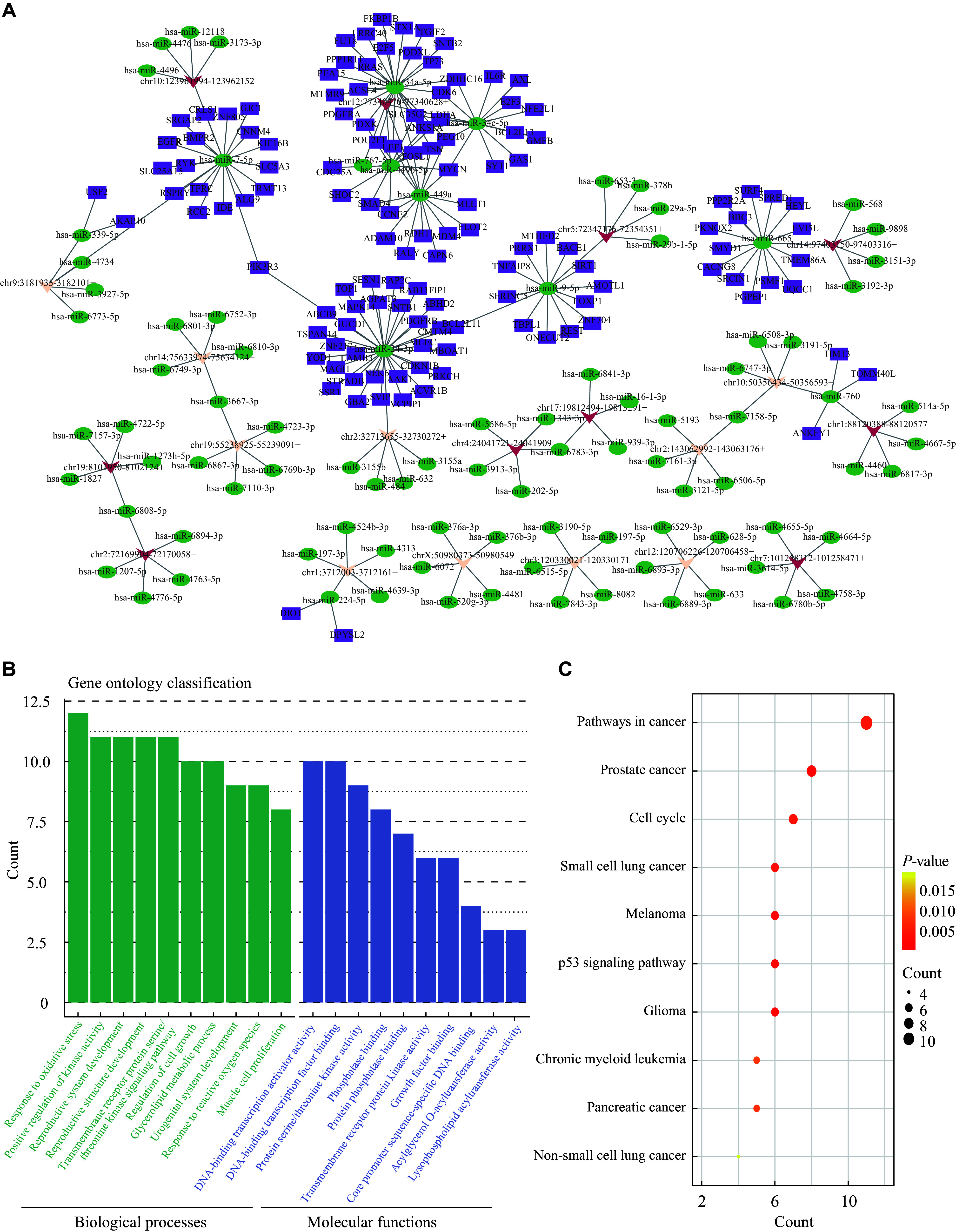
The interaction network among circRNAs, miRNAs, and mRNAs. A: The circRNA-miRNA-mRNA interaction network diagram of the top 10 dysregulated circRNAs. Red and yellow triangles represent upregulated and downregulated circRNAs, respectively. The top five miRNAs regulated by each circRNA are indicated by green circles. The purple squares represent the targeted mRNAs of the top five miRNAs, as predicted by four software programs, including TargetScan, miRwalk, miRDB, and miRTarBase. B: GO enrichment analysis of the predicted mRNAs, with the top 10 GO terms shown in biological processes and molecular functions. C: The top 10 KEGG pathways correlated with the predicted mRNAs.

Currently, research on the mechanisms of disease-related mRNAs has shifted to circRNAs, particularly their roles in the crosstalk among circRNAs, miRNAs, and mRNAs. As expected, a total of 56254 miRNAs were found to have binding sites on the sequences of their corresponding circRNAs. To further investigate the underlying role of circRNAs in exosomes derived from the PTC patients with LNM, the top 10 upregulated and top 10 downregulated circRNAs were selected for the construction of ceRNA regulatory networks (***Supplementary Table 2***, available online). The top five related miRNAs were shown in the regulatory networks based on the context score and free energy calculated by both the TargetScan and miRanda software. Subsequently, we predicted potential target mRNAs of these miRNAs by intersecting the results from four prediction programs, and screened a total of 127 predicted genes (***Fig. 6A***). Functional annotation analysis revealed a total of 25 enriched GO terms in BP and 13 terms in MF for these predicted target genes (***Fig. 6B***). Additionally, the KEGG pathway analysis revealed 10 signaling pathways related to the predicted regulatory genes, some of which have been reported to be closely associated with PTC progression, such as mitogen-activated protein kinase (MAPK) and p53 signaling pathways (***Fig. 6C***).

Next, we examined the transcript levels of these intersecting top five miRNAs in PTC by reported studies and identified seven miRNAs that exhibited an inverse expression pattern, compared with their corresponding circRNAs. We further investigated the role of the 127 predicted target genes in PTC by reported studies and identified 14 candidate genes linked with LNM in PTC patients (***Supplementary Table 3***, available online). Together, these circRNA/miRNA/mRNA axes, including six circRNAs, seven miRNAs, and 14 target genes, provided important clues for the mechanisms of LNM in PTC.

### Validation of the screened circRNA-miRNA-mRNA networks

**Figure 7:**
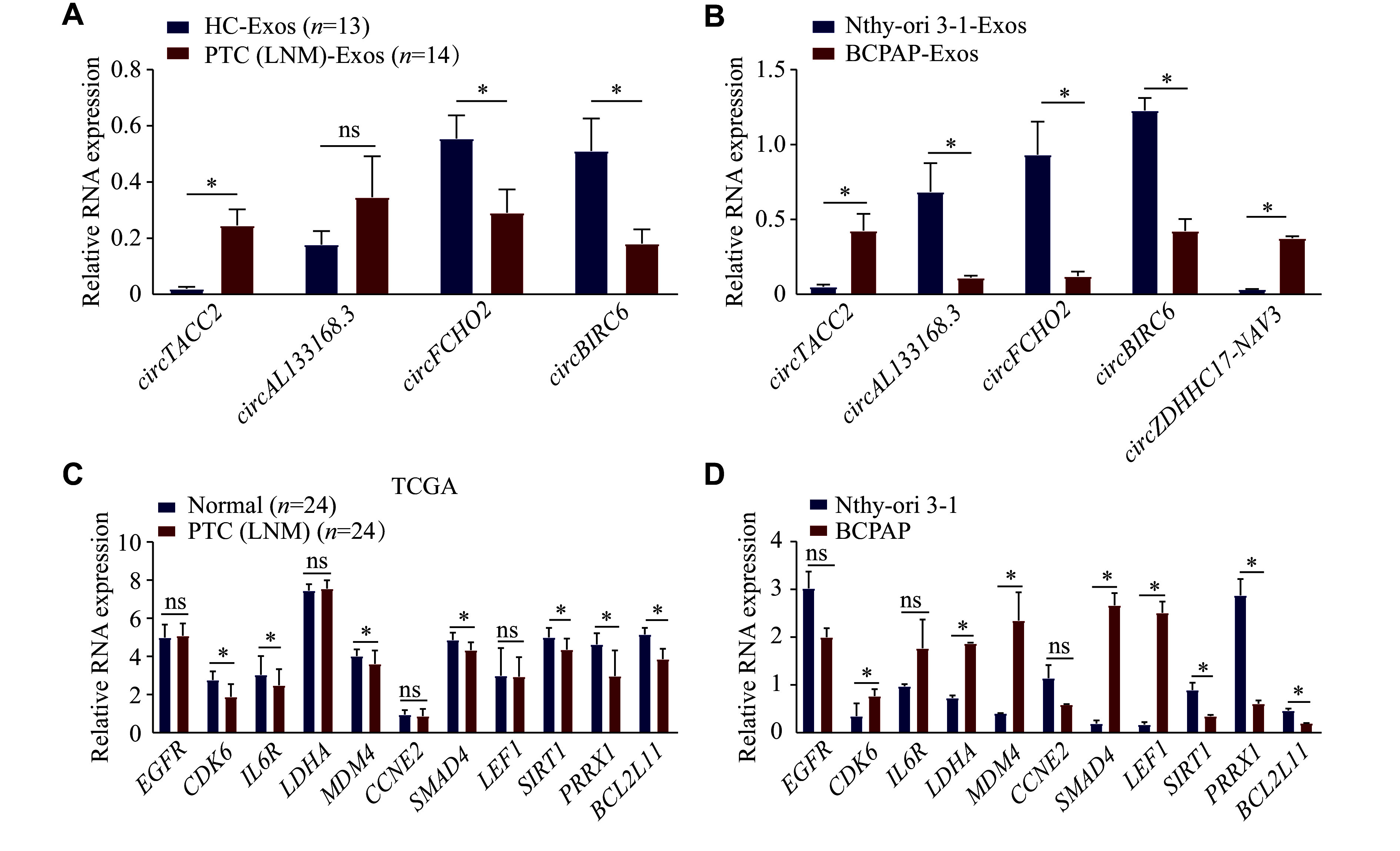
Validation of the screened circRNA-miRNA-mRNA networks. A: Serum exosomes were isolated from 14 papillary thyroid carcinoma (PTC) patients with lymph node metastasis (LNM) and 13 healthy controls. The transcript levels of *circTACC2*, *circAL133168.3*, *circFCHO2*, and *circBIRC6* were analyzed by quantitative reverse transcription-PCR (qRT-PCR). B: The transcript levels of *circTACC2*, *circAL133168.3*, *circFCHO2*, *circBIRC6*, and *circZDHHC17-NAV3* in exosomes from Nthy-ori 3-1 and BCPAP cell lines were analyzed by qRT-PCR. C and D: The transcript levels of 11 potential regulatory genes correlated with *circTACC2*, *circFCHO2*, *circBIRC6*, and *circZDHHC17-NAV3* were analyzed using The Cancer Genome Atlas database (TCGA) and validated in Nthy-ori 3-1 and BCPAP cell lines, respectively. Data are presented as mean ± standard deviation from three independent experiments. Statistical analyses were performed by Student's *t*-test. ^*^P < 0.05. Abbreviation: ns, not significant.

To further investigate the specific role of circRNAs, we selected the six screened circRNAs for preliminary validation by expanding the sample size and cell lines. The BCPAP cell line was selected for validation because it was established from the tumor tissues of a female patient with metastatic PTC^[20]^. Firstly, we renamed the selected circRNAs based on their source genes. Our data showed that the expression levels of three out of four circRNAs in serum exosomes and four out of five circRNAs in cell-derived exosomes were consistent with the sequencing data in serum exosomes (***Fig. 7A***) and in cell line-derived exosomes (***Fig. 7B***), respectively. However, *circLRRC47* was not amplified by appropriate primers, and *circZDHHC17-NAV3* was not detected in serum exosomes because of its low expression. Intriguingly, compared with that of the controls, *circFCHO2* expression was attenuated in serum exosomes from PTC patients with LNM and in BCPAP cell-derived exosomes, which was contrary to the sequencing results.

Then, a total of 24 paired tissue samples from PTC patients with LNM were screened in the TCGA database. The expression levels of 11 potential regulatory protein-coding genes of *circTACC2*, *circFCHO2*, *circBIRC6*, and *circZDHHC17-NAV3* were analyzed in the TCGA database and verified in Nthy-ori 3-1 and BCPAP cell lines. The significant expression levels of *SIRT1*, *PRRX1*, and *BCL2L11* showed the same trend in both the TCGA database and cell lines (***Fig. 7C*** and ***7D***). In contrast, the expression trend of the other eight potentially regulated genes was inconsistent between the TCGA database and cell lines.

**Figure 8:**
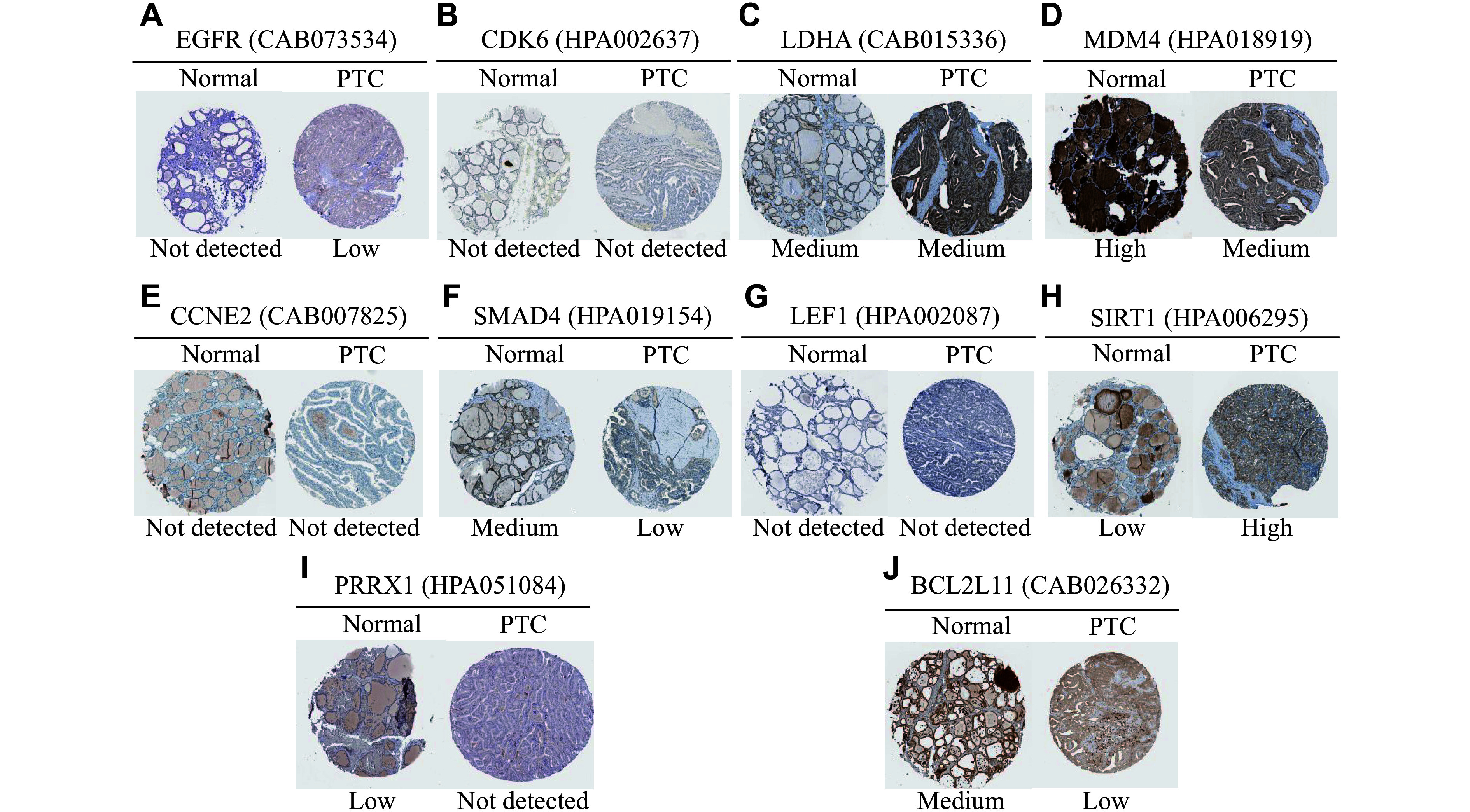
The protein expression of potential regulatory genes in the circRNA-miRNA-mRNA networks. Immunohistochemical staining of EGFR (A), CDK6 (B), LDHA (C), MDM4 (D), CCNE2 (E), SMAD4 (F), LEF1 (G), SIRT1 (H), PRRX1 (I), and BCL2L11 (J) in papillary thyroid carcinoma and normal thyroid tissues. The immunohistochemical images were obtained from the Human Protein Atlas (HPA) database. No data were available for IL6R in the HPA database. The URLs of the images are shown in ***Supplementary Table 4*** (available online).

Furthermore, immunohistochemical staining data derived from the HPA database were analyzed to determine the protein levels of the potentially regulated genes. As shown in ***Fig. 8***, heightened staining intensity of EGFR and SIRT1, as well as attenuated staining intensity of MDM4, SMAD4, and BCL2L11, were observed in PTC tissues, compared with normal thyroid tissues. Notably, strong staining for EGFR was also observed in other PTC samples (data not shown). Based on the ceRNA network and the expression trends of miRNAs in PTC, the *circTACC2*-miR-7-*EGFR* and *circBIRC6*-miR-24-3p-*BCL2L11* axes were identified as potentially involved in PTC with LNM through preliminary verification.

### PPI network module analysis of dysregulated circRNAs

**Figure 9:**
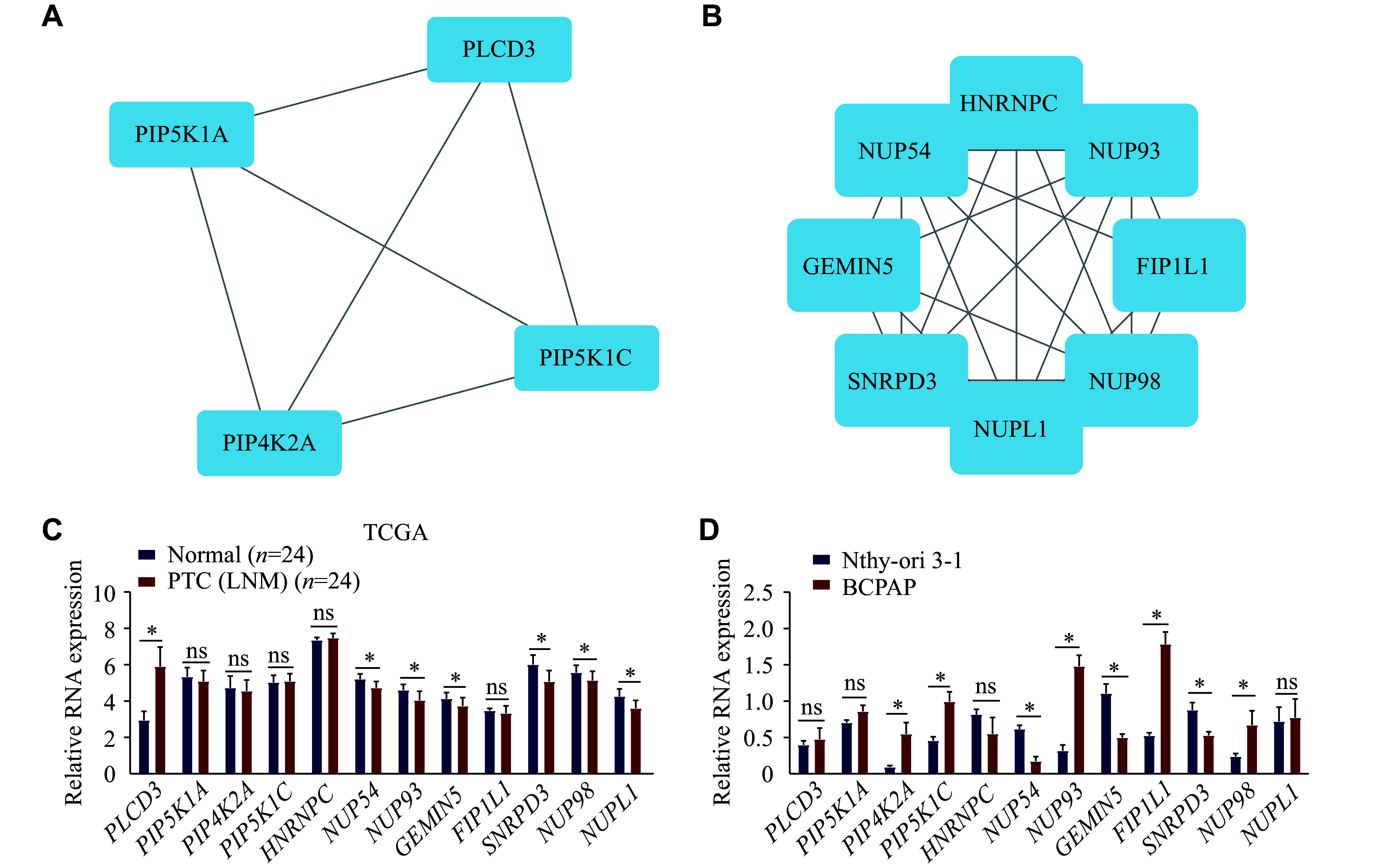
Protein-protein interaction (PPI) network module analysis of dysregulated circRNAs. Cytoscape was used to construct the PPI network. The MCODE algorithm was applied to analyze the host genes of dysregulated circRNAs. A and B: Top module of host genes correlated with upregulated (A) or downregulated (B) circRNAs in the PPI network. C and D: Transcript levels of 12 hub genes were analyzed using The Cancer Genome Atlas database (C) and validated in Nthy-ori 3-1 and BCPAP cell lines (D), respectively. Data are presented as mean ± standard deviation from three independent experiments. Statistical analyses were performed by Student's *t*-test. ^*^P < 0.05. Abbreviations: PLCD3, phospholipase C delta 3; PIP5K1A, phosphatidylinositol-4-phosphate 5-kinase type 1 alpha; PIP4K2A, phosphatidylinositol-5-phosphate 4-kinase type 2 alpha; PIP5K1C, phosphatidylinositol-4-phosphate 5-kinase type 1 gamma; HNRNPC, heterogeneous nuclear ribonucleoprotein C; NUP54, nucleoporin 54; NUP93, nucleoporin 93; GEMIN5, gem nuclear organelle associated protein 5; FIP1L1, factor interacting with PAPOLA and CPSF1; SNRPD3, small nuclear ribonucleoprotein D3 polypeptide; NUP98, nucleoporin 98; NUPL1, nucleoporin like 1; ns, not significant.

**Figure 10:**
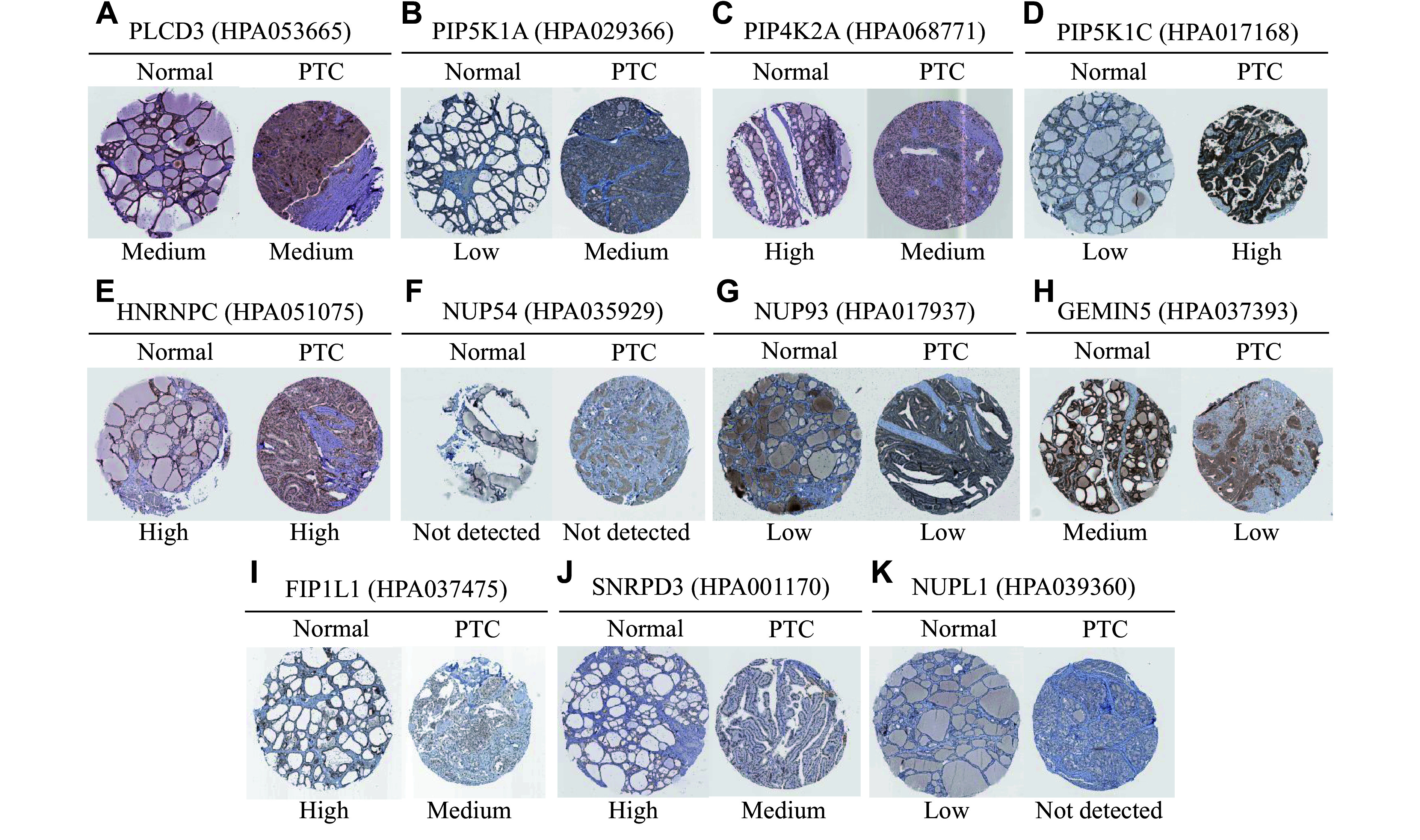
The protein expression levels of hub genes regulated by dysregulated circRNAs. Immunohistochemical staining of PLCD3 (A), PIP5K1A (B), PIP4K2A (C), PIP5K1C (D), HNRNPC (E), NUP54 (F), NUP93 (G), GEMIN5 (H), FIP1L1 (I), SNRPD3 (J), and NUPL1 (K) in papillary thyroid carcinoma and normal thyroid tissues. The immunohistochemical images were obtained from the Human Protein Atlas (HPA) database. No data were available for NUP98 in the HPA database. The URLs of the images are shown in ***Supplementary Table 4*** (available online). Abbreviations: PLCD3, phospholipase C delta 3; PIP5K1A, phosphatidylinositol-4-phosphate 5-kinase type 1 alpha; PIP4K2A, phosphatidylinositol-5-phosphate 4-kinase type 2 alpha; PIP5K1C, phosphatidylinositol-4-phosphate 5-kinase type 1 gamma; HNRNPC, heterogeneous nuclear ribonucleoprotein C; NUP54, nucleoporin 54; NUP93, nucleoporin 93; GEMIN5, gem nuclear organelle associated protein 5; FIP1L1, factor interacting with PAPOLA and CPSF1; SNRPD3, small nuclear ribonucleoprotein D3 polypeptide; NUP98, nucleoporin 98; NUPL1, nucleoporin like 1.

A total of 600 upregulated circRNA-derived host genes and 1761 downregulated circRNA-derived host genes were imported into the PPI network. The nodes with a PPI score > 0.9 were screened to establish the PPI networks. For the upregulated circRNAs, 354 nodes were identified, of which 293 nodes exhibited interaction relationships (***Supplementary Fig. 1A***, available online). The top modules of host genes correlated with upregulated circRNAs in the PPI network were screened using the MCODE algorithm (***Fig. 9A***). For the downregulated circRNAs, 1043 nodes were identified, with 986 nodes exhibiting interaction relationships (***Supplementary Fig. 1B***, available online). The top modules of host genes correlated with downregulated circRNAs in the PPI network were also screened using the MCODE algorithm (***Fig. 9B***). Finally, we identified 12 hub genes with a high degree of connectivity, including four hub genes related to upregulated circRNAs and eight hub genes related to downregulated circRNAs. To further investigate the role of these hub genes in PTC, we examined their transcript levels in the TCGA database and PTC cell lines. Our data revealed that the mRNA levels of *NUP54*, *GEMIN5*, and *SNRPD3* were decreased in both tissues from PTC patients with LNM and BCPAP cells (***Fig. 9C*** and ***9D***). The expression levels of other hub genes were inconsistent between the TCGA database and PTC cell lines. However, only *GEMIN5* and *SNRPD3* exhibited the same attenuated expression trend in PTC tissues, compared with normal thyroid tissues in the HPA database (***Fig. 10***). These findings preliminarily validate the potential regulation of the screened hub genes by the dysregulated circRNAs in PTC with LNM.

## Discussion

PTC may exhibit aggressive behavior, usually preceded by LNM^[21]^. According to previous studies, the incidence of LNM in PTC ranged from 30% to 80%^[22–23]^. LNM is a significant risk factor for poor prognosis in PTC patients^[6]^. However, the mechanisms underlying LNM in PTC remain unclear. Recently, exosomal circRNAs have attracted attention because of their unique function in material communication of TME. Dai *et al*^[24]^ found that *hsa_circ_0082002* and *hsa_circ_0003863* in exosomes may contribute to the diagnosis of PTC. Lin *et al*^[25]^ demonstrated that exosomal *hsa_circ_007293* shared functional properties with the miR-653/paired box 6 axis and promoted the progression of PTC. Despite these findings, the molecular mechanisms regulating PTC with LNM by exosomal circRNAs still require further clarification. In the current study, we characterized the expression profiles of circRNAs in serum exosomes and identified 2877 significantly dysregulated circRNAs in PTC patients with LNM. Notably, the majority of these dysregulated circRNAs were novel circRNAs that had not been identified or listed in the circBase or Circ2Traits databases. This discrepancy may be attributed to the fact that these two databases primarily include exonic circRNAs (ecircRNAs), while the circRNAs identified in the current study were mainly intronic circRNAs. Given the release of exosomes as a critical tool for cellular communication in the TME^[26]^, these dysregulated circRNAs may serve as key components of exosomes, providing a basis for the mechanistic research of LNM in PTC.

We subsequently investigated the potential functions of the dysregulated circRNAs through bioinformatics analyses of their host genes. In the GO terms, cellular components are fundamental elements of tumor biological behavior, and the enriched biological molecules, such as p53 and GTPase, have been reported to play critical roles in the progression of PTC^[27–28]^. In addition, 91 signaling pathways correlated with the host genes of dysregulated circRNAs were identified. Among these pathways, the glucagon signaling pathway, the most relevant pathway for upregulated circRNAs, promotes PTC invasion and migration by mediating the epithelial-mesenchymal transition and p38/extracellular-regulated kinase (ERK) pathways^[29]^. Other relevant pathways correlated with upregulated circRNAs, such as the WNT and ERBB signaling pathways, are known to be involved in LNM in PTC patients^[17–18]^. The phospholipase D signaling pathway was the most relevant pathway for downregulated circRNAs, which synergistically activates signal transducer and activator of transcription 3 (STAT3) by directly interacting with the *RET*/*PTC* gene^[30]^. Additionally, calmodulin-dependent kinaseⅡ (CaMKⅡ) contributes to the activation of ERK cascade, thereby promoting the progression of PTC^[19]^. The HIF-1 signaling pathway facilitates the aggressive and progressive metastatic form of PTC by reprogramming the glucose/iodine metabolic program in hypoxic conditions^[31]^. Furthermore, MAPK is present in various signaling pathways and is broadly known to be involved in LNM of PTC^[32]^. The PI3K/AKT, another important signaling pathway closely related to multiple KEGG pathways, functions in thyroid tumorigenesis and progression by enhancing protein synthesis and overall reprogramming of cancer cell metabolism^[33–34]^. Taken together, these enriched signaling pathways suggest that dysregulated exosomal circRNAs may be involved in the LNM of PTC and serve as novel research targets for understanding the mechanisms of LNM in PTC.

As an important regulatory mechanism for circRNAs, the crosstalk among circRNA, miRNA, and mRNA has been widely investigated. There are miRNA binding sites in the circRNA sequence, and miRNA may target the 3′ UTR of mRNA. The exact mechanism is that circRNA acts as a sponge for miRNA, resulting in decreased miRNA expression, thereby releasing the suppression of target genes by miRNAs^[35]^. ciRS-7 was the first identified circRNA in humans and mice, which promoted the expression levels of miR-7 target genes by sponging miR-7^[36]^. In PTC, the functional mechanism of circRNA primarily involves miRNA sponging^[15]^. For example, the upregulated *hsa_circ_0058124* identified in PTC patients promoted the invasion and metastasis of tumor cells by targeting miR-218-5p, thereby terminating the inhibitory effect of *NUMB*^[37]^. Similarly, *circTIAM1* was demonstrated to activate the HNRNPA1 signaling pathway and promote the progression of PTC by sponging miR-646^[38]^. Based on the ceRNA network and the key role of exosomes as a "bridge" in TME communication, we speculated that these dysregulated exosomal circRNAs might influence LNM in PTC by sponging miRNAs. Hence, we selected the top 10 dysregulated circRNAs for their miRNA and mRNA target prediction. Subsequent GO and KEGG enrichment analyses of 127 predicted target genes revealed a significant enrichment in multiple PTC-related signaling pathways, including p53, MAPK, and PI3K/AKT pathways. The deletion of *P53*, a classic tumor suppressor gene, is one of the most characteristic genetic changes contributing to poorly differentiated thyroid malignancies^[39]^. In PTC, the activation of MAPK and PI3K/AKT signaling pathways leads to abnormal proliferation and metastasis of PTC cells^[40–41]^.

Through comprehensive analysis, we screened 14 predicted circRNA/miRNA/mRNA axes potentially correlated with LNM in PTC, including four upregulated and two downregulated circRNAs. Validation in expanded samples and cell lines as well as database analysis revealed that the *circTACC2*-miR-7-EGFR and *circBIRC6*-miR-24-3p-BCL2L11 axes might be involved in LNM of PTC. EGFR is associated with the progression of PTC, triggering a PI3K-AKT signal cascade to promote the invasion and metastasis of tumor cells^[42]^. However, the mechanisms underlying elevated EGFR levels in PTC remain poorly understood. In the current study, we screened a novel upregulated circRNA, *circTACC2*, which might promote EGFR expression by interacting with miR-7 in PTC patients with LNM. Notably, miR-7 expression has been reported to be decreased in PTC specimens, where it inhibits the progression of PTC^[43]^. Another downregulated ecircRNA, *circBIRC6*, may facilitate the progression of PTC by regulating the miR-24-3p/*BCL2L11* axis. BCL2L11, a member of the BCL2 family, functions as a tumor suppressor by regulating cell apoptosis^[44]^. Moreover, as commonly observed to be downregulated in PTC patients, BCL2L11 has been reported to be induced after treatment with Src and MEK1/2 inhibitors, thereby enhancing apoptosis^[45]^. Together, these ceRNA networks underscore the significant role of exosomal circRNAs in the progression of PTC.

Moreover, we screened 12 hub genes (*i.e.*, *PLCD3*, *PIP5K1A*, *PIP4K2A*, *PIP5K1C*, *HNRNPC*, *NUP54*, *NUP93*, *GEMIN5*, *FIP1L1*, *SNRPD3*, *NUP98*, and *NUPL1*) with high connectivity from PPI networks by bioinformatics analysis. Among them, *PLCD3* has been identified as an oncogenic gene in PTC, with its regulatory mechanism mediated through the *has_circ_0003747*/miR-338-3p/*PLCD3* axis^[46]^. Lin *et al*^[47]^ also found that PLCD3 expression was correlated with LNM and disease stage in thyroid cancer *via* the Hippo signaling pathway. Thus, the elevated levels of PLCD3 may play a critical role in identifying LNM of PTC. Additionally, PLCD3 is a *BRAF*^*V600E*^-associated biomarker with both prognostic and diagnostic significance in PTC patients^[48]^. Our data showed that *PLCD3* mRNA expression levels were increased in PTC patients with LNM. However, immunohistochemical staining intensity was moderate in both PTC and normal thyroid tissues from the HPA database. One possible explanation for this discrepancy is that the immunohistochemical staining may be too strong, which requires further verification. Regarding other hub genes, *GEMIN5* expression was upregulated in low-metastatic breast cancer cells, and the siRNA-mediated reduction of *GEMIN5* expression increased the motility of tumor cells by affecting the expression of spliceosomal proteins^[49]^, suggesting that the depressed GEMIN5 expression may be associated with LNM in PTC. In addition, *SNRPD3*, as a co-factor for MYCN oncogenesis, participates in maintaining the fidelity and balance of alternative splicing events in neuroblastoma cells^[50]^. However, the role of attenuated SNRPD3 expression in PTC patients with LNM remains poorly understood.

The findings of the current study provide novel insights into potential candidate genes for future research. However, some limitations should be acknowledged. First, the sample size for verification was small, which requires future verification by expanding the sample size. Second, the detailed mechanisms of certain circRNAs in PTC patients with LNM remain unexplored.

In summary, the current study provides the first expression profile of exosomal circRNAs and elucidates the connection between circRNAs, miRNAs, and mRNAs in PTC patients with LNM. The identified exosomal circRNAs and their potential functions may shed light on new molecular mechanisms underlying the LNM in PTC and serve as potential biomarkers in PTC patients with LNM.

## SUPPLEMENTARY DATA

Supplementary data to this article can be found online.
