# Supplementary material for: Expression profiling and bioinformatics analysis of serum exosomal circular RNAs in lymph node metastasis of papillary thyroid carcinoma
Source: J Biomed Res. 2024 May 30;39(2):155–70. doi: 10.7555/JBR.37.20230304 (PMC11982685; doi:10.7555/JBR.37.20230304)
Supplement: Supplementary file 1 — Supplementary data to this article can be found online. [file jbr-39-2-155-S1.pdf]

Available online at www.jbr-pub.org.cn

Open Access at PubMed Central

JBR

Journal of Biomedical Research, 2025 39(2): 1–4

Supplementary Data

Expression profiling and bioinformatics analysis of serum exosomal circular RNAs in lymph node metastasis of papillary thyroid carcinoma

Huiyong Peng[1,2,3], Zhangwei Zhu[4], Jie Xing[3], Qian Xu[4], Changfeng Man[5], Shengjun Wang[6], Yingzhao Liu[4,✉], Zhengdong Zhang[1,2,✉]

[1]*Department of Environmental Genomics, Jiangsu Key Laboratory of Cancer Biomarkers, Prevention and Treatment, Collaborative Innovation Center for Cancer Personalized Medicine, School of Public Health, Nanjing Medical University, Nanjing, Jiangsu 211166, China;*
[2]*Department of Genetic Toxicology, the Key Laboratory of Modern Toxicology of Ministry of Education, Center for Global Health, School of Public Health, Nanjing Medical University, Nanjing, Jiangsu 211166, China;*
[3]*Department of Laboratory Medicine, Zhenjiang Medical School of Nanjing Medical University, Zhenjiang, Jiangsu 212002, China;*
[4]*Department of Endocrinology, Zhenjiang Medical School of Nanjing Medical University, Zhenjiang, Jiangsu 212002, China;*
[5]*Department of Oncology, Zhenjiang Medical School of Nanjing Medical University, Zhenjiang, Jiangsu 212002, China;*
[6]*Department of Laboratory Medicine, the Affiliated Hospital of Jiangsu University, Zhenjiang, Jiangsu 212008, China.*

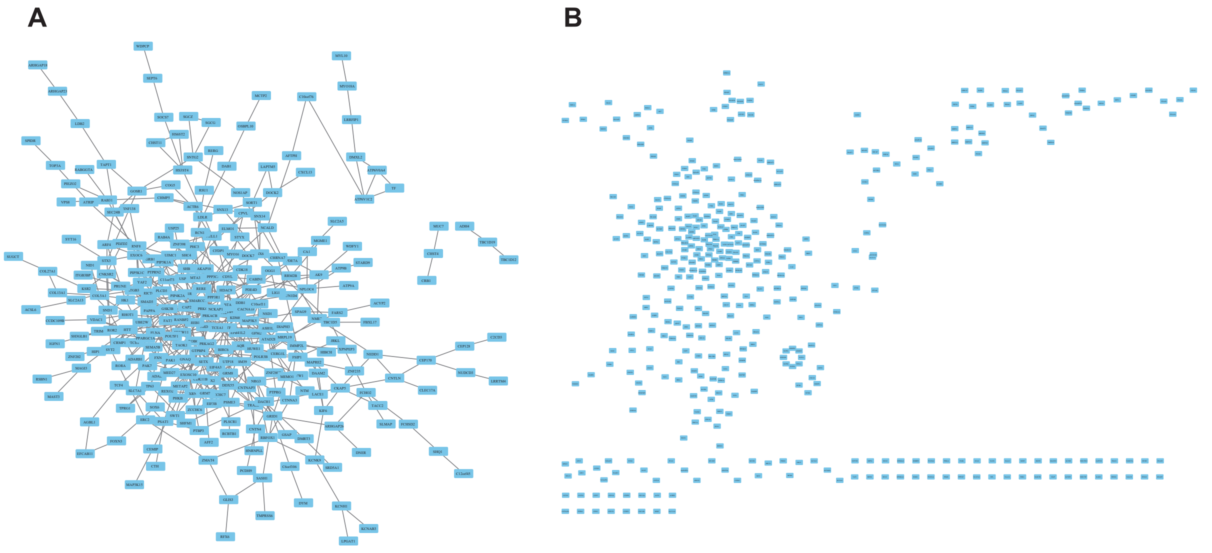

Supplementary Fig. 1 **Protein-protein interaction (PPI) network module analysis of dysregulated circRNAs.** Cytoscape was used to construct the PPI network. A and B: The PPI network of the host genes correlated with upregulated (A) or downregulated (B) circRNAs.

✉Corresponding authors: Yingzhao Liu, Department of Endocrinology, Zhenjiang Medical School of Nanjing Medical University, 8 Dianli Road, Zhenjiang, Jiangsu 212002, China. E-mail: zjliuyingzhao@126.com; Zhengdong Zhang, Department of Environmental Genomics, School of Public Health, Nanjing Medical University, 101 Longmian Avenue, Nanjing, Jiangsu 211166, China. E-mail: drzdzhang@njmu.edu.cn.

Received: 21 December 2023; Revised: 28 April 2024; Accepted: 23 May 2024; Published online: 30 May 2024

CLC number: R736.1, Document code: A

The authors reported no conflict of interests.

This is an open access article under the Creative Commons Attribution (CC BY 4.0) license, which permits others to distribute, remix, adapt and build upon this work, for commercial use, provided the original work is properly cited.

© 2025 by Journal of Biomedical Research.

https://doi.org/10.7555/JBR.37.20230304

Supplementary Table 1 **The primer sequences for verified circRNAs and potential regulatory genes**

Genes	Types	Primer sequences
circTACC2	F	5′-TTCCCTGTTTTGGTCAGTACA-3′
	R	5′-GGAAATCCCAGGATGATGGCA-3′
circAL133168.3	F	5′-ACAGATTGGACCAATGCGATAAT-3′
	R	5′-TCTCATCCTAGGCCTGACCA-3′
circFCHO2	F	5′-GACCCCCTATTTGGACCATCTC-3′
	R	5′-TGTTTCTGGTTTAATACTGTAGCCT-3′
circBIRC6	F	5′-GGCTTGGTGTGACCTTACCA-3′
	R	5′-AGAATTCAGGCTCAGTGTGAG-3′
circZDHHC17-NAV3	F	5′-CCTGGATCAGCTAGGGTAACACT-3′
	R	5′-CGGAGGCATACGTGCACAC-3′
circLRRC47	F	5′-GCCACATGCTGTGATGTGGTAC-3′
	R	5′-GACAGGTCTCTACGCTCGATCTAC-3′
ACTB	F	5′-CACGAAACTACCTTCAACTCC-3′
	R	5′-CATACTCCTGCTTGCTGATC-3′
EGFR	F	5′-CAGATCGCAAAGGGCATGAA-3′
	R	5′-TTGCCTCCTTCTGCATGGTA-3′
CDK6	F	5′-ATAGATGCCAGGCAGAGACC-3′
	R	5′-ACTGCCTGTTCCCACTACTC-3′
IL6R	F	5′-CTGAGGGTGAGTGGGTGAAT-3′
	R	5′-CCCTCTCCTCTCCTTCCTCT-3′
LDHA	F	5′-CTCTGAAGACTCTGCACCCA-3′
	R	5′-ATAGCCCAGGATGTGTAGCC-3′
MDM4	F	5′-TGATCAGCAGGAGCAGCATA-3′
	R	5′-AGAGAGGGCTTGGGTCTTTC-3′
CCNE2	F	5′-ATACTGACTGCTGCTGCCTT-3′
	R	5′-AGTCTTCAGCTTCACTGGACT-3′
SMAD4	F	5′-GACCCTTCTGGAGGAGATCG-3′
	R	5′-ACCTTGCTCTCTCAATGGCT-3′
LEF1	F	5′-CAGATCGCAAAGGGCATGAA-3′
	R	5′-TTGCCTCCTTCTGCATGGTA-3′
SIRT1	F	5′-TATGCTCGCCTTGCTGTAGA-3′
	R	5′-TGGCTGGAATTGTCCAGGAT-3′
PRRX1	F	5′-GAGCGGACACACTATCCTGA-3′
	R	5′-CTCATTCCTGCGGAACTTGG-3′
BCL2L11	F	5′-AGAGTTGCGGCGTATTGGAGAC-3′
	R	5′-GGTGGTCTTCGGCTGCTTGG-3′
PLCD3	F	5′-ACTACGTGCTCAACAATGGC-3′
	R	5′-TACCCTTGCTTTAGGCTGCT-3′
PIP5K1A	F	5′-CTATGCACCTGTTGCCTTCC-3′
	R	5′-TCATCGTCGCTGGACACATA-3′
PIP4K2A	F	5′-CTACAGTCTGCTGGTGGGAA-3′
	R	5′-CGTTCTCCTCACACTCCACT-3′
PIP5K1C	F	5′-CTCAAGGGCTCCACCTACAA-3′
	R	5′-TGCATGAAGTCCAGGTCCTT-3′
HNRNPC	F	5′-AACAGAACACCCTTCTCCGT-3′
	R	5′-CTACAGCCCGAGCAATAGGA-3′
NUP54	F	5′-ACTGGTTTGGGAACTGGACT-3′
	R	5′-GTTGGACTGGGTAGGAGCTT-3′
NUP93	F	5′-GTGTTCTGCCTTCCTCCTCT-3′
	R	5′-CTCTGCAGGGCRCATGTTTC-3′
GEMIN5	F	5′-AGGCGTGAAGTCCAAGGTTA-3′
	R	5′-GGAGGCTTGTTGGAGTAGGT-3′
FIP1L1	F	5′-CTTCCACCGAGCAGAAACAG-3′
	R	5′-TGATTCGGCCCTCCCATATC-3′
SNRPD3	F	5′-CAGAGCCGAACTCTCTTCCT-3′
	R	5′-CCACTCGGCCATCTCTGTAT-3′
NUP98	F	5′-GTGACATGTCTGTGGGAAGC-3′
	R	5′-GCAGGCAAATCCAGACAGAG-3′
NUPL1	F	5′-CATGTCCACAGGGTTCTCCT-3′
	R	5′-CCCACAGAAGGGTTGCTAGA-3′

Abbreviations: F, forward; R, reverse.

Supplementary Table 2 **Top 10 significantly dysregulated circRNAs in exosomes of PTC patients with LNM, compared with those of healthy controls**

circRNA ID	log_2FC	*P*	Regulation	Chromosomes	Strand	Catalogs
chr7:101258312–101258471	9.953	0.017	Up	chr7	+	Antisense circRNA
chr5:72347176–72354351	9.86	0.018	Up	chr5	+	ecircRNA
chr14:97403150–97403316	9.72	0.019	Up	chr14	–	Antisense circRNA
chr12:77340470–77340628	9.43	0.021	Up	chr12	+	Intergenic circRNA
chr10:123961994–123962152	9.39	0.021	Up	chr10	+	Intergenic circRNA
chr2:72169904–72170058	9.38	0.021	Up	chr2	–	Antisense circRNA
chr1:88120388–88120577	9.23	0.022	Up	chr1	–	ciRNA
chr4:24041721–24041909	9.14	0.023	Up	chr4	–	ciRNA
chr17:19812494–19813291	9.13	0.023	Up	chr17	–	ecircRNA
chr19:8101950–8102124	9.04	0.023	Up	chr19	+	ciRNA
chr9:3181935–3182101	−9.91	0.014	Down	chr9	+	ciRNA
chr12:120706226–120706458	−9.80	0.015	Down	chr12	–	Intergenic circRNA
chr2:32713655–32730272	−9.80	0.015	Down	chr2	+	ecircRNA
chr14:75633974–75634124	−9.55	0.016	Down	chr14	–	ciRNA
chrX:50980373–50980549	−9.52	0.029	Down	chrX	–	Intergenic circRNA
chr3:120330021–120330171	−9.48	0.016	Down	chr3	–	Intergenic circRNA
chr2:143062992–143063176	−9.40	0.017	Down	chr2	+	ciRNA
chr10:50356434–50356593	−9.37	0.017	Down	chr10	–	Antisense circRNA
chr1:3712003–3712161	−9.37	0.017	Down	chr1	–	ciRNA
chr19:55238925–55239091	−9.35	0.017	Down	chr19	+	ciRNA

Abbreviation: FC, fold change; ecircRNA, exonic circRNA; ciRNA, intronic circRNA.

Supplementary Table 3 **Identification of circRNA-miRNA-mRNA networks**

circRNAs	Source genes	Interacting miRNAs	Targeting mRNAs
chr10:123961994–123962152+	*TACC2*	hsa-miR-7-5p	*EGFR*
chr12:77340470–77340628+	*ZDHHC17-NAV3*	hsa-miR-34c-5p	*CDK6*
			IL6R
			LDHA
		hsa-miR-449a	*MDM4*
			CCNE2
			SMAD4
			LEF1
chr14:97403150–97403316−	*AL133168.3*	hsa-miR-665	*PPP2R2A*
			BBC3
chr5:72347176–72354351+	*FCHO2*	hsa-miR-9-5p	*SIRT1*
			PRRX1
chr1:3712003–3712161−	*LRRC47*	hsa-miR-224-5p	*DIO1*
chr2:32713655–32730272+	*BIRC6*	hsa-miR-24-3p	*BCL2L11*

Abbreviations: EGFR, epidermal growth factor receptor; CDK6, cyclin dependent kinase 6; IL6R, interleukin 6 receptor; LDHA, lactate dehydrogenase A; MDM4, MDM4 regulator of p53; CCNE2, cyclin E2; SMAD4, SMAD family member 4; LEF1, lymphoid enhancer binding factor 1; PPP2R2A, protein phosphatase 2 regulatory subunit Balpha; BBC3, BCL2 binding component 3; SIRT1, sirtuin 1; PRRX1, paired related homeobox 1; DIO1, iodothyronine deiodinase 1; BCL2L11, BCL2 like 11.

Supplementary Table 4 **The URLs of the images obtained from the HPA database**

Proteins	Tissue types	URLs
EGFR	PTC	https://www.proteinatlas.org/ENSG00000146648-EGFR/pathology/thyroid+cancer
	Normal	https://www.proteinatlas.org/ENSG00000146648-EGFR/tissue/thyroid+gland
CDK6	PTC	https://www.proteinatlas.org/ENSG00000105810-CDK6/pathology/thyroid+cancer
	Normal	https://www.proteinatlas.org/ENSG00000105810-CDK6/tissue/thyroid+gland
LDHA	PTC	https://www.proteinatlas.org/ENSG00000134333-LDHA/pathology/thyroid+cancer
	Normal	https://www.proteinatlas.org/ENSG00000134333-LDHA/tissue/thyroid+gland
MDM4	PTC	https://www.proteinatlas.org/ENSG00000198625-MDM4/pathology/thyroid+cancer
	Normal	https://www.proteinatlas.org/ENSG00000198625-MDM4/tissue/thyroid+gland
CCNE2	PTC	https://www.proteinatlas.org/ENSG00000175305-CCNE2/pathology/thyroid+cancer
	Normal	https://www.proteinatlas.org/ENSG00000175305-CCNE2/tissue/thyroid+gland
SMAD4	PTC	https://www.proteinatlas.org/ENSG00000141646-SMAD4/pathology/thyroid+cancer
	Normal	https://www.proteinatlas.org/ENSG00000141646-SMAD4/tissue/thyroid+gland
LEF1	PTC	https://www.proteinatlas.org/ENSG00000138795-LEF1/pathology/thyroid+cancer
	Normal	https://www.proteinatlas.org/ENSG00000138795-LEF1/tissue/thyroid+gland
SIRT1	PTC	https://www.proteinatlas.org/ENSG00000096717-SIRT1/pathology/thyroid+cancer
	Normal	https://www.proteinatlas.org/ENSG00000096717-SIRT1/tissue/thyroid+gland
PRRX1	PTC	https://www.proteinatlas.org/ENSG00000116132-PRRX1/pathology/thyroid+cancer
	Normal	https://www.proteinatlas.org/ENSG00000116132-PRRX1/tissue/thyroid+gland
BCL2L11	PTC	https://www.proteinatlas.org/ENSG00000153094-BCL2L11/pathology/thyroid+cancer
	Normal	https://www.proteinatlas.org/ENSG00000153094-BCL2L11/tissue/thyroid+gland
PLCD3	PTC	https://www.proteinatlas.org/ENSG00000161714-PLCD3/pathology/thyroid+cancer
	Normal	https://www.proteinatlas.org/ENSG00000161714-PLCD3/tissue/thyroid+gland
PIP5K1A	PTC	https://www.proteinatlas.org/ENSG00000143398-PIP5K1A/pathology/thyroid+cancer
	Normal	https://www.proteinatlas.org/ENSG00000143398-PIP5K1A /tissue/thyroid+gland
PIP4K2A	PTC	https://www.proteinatlas.org/ENSG00000150867-PIP4K2A/pathology/thyroid+cancer
	Normal	https://www.proteinatlas.org/ENSG00000150867-PIP4K2A/tissue/thyroid+gland
PIP5K1C	PTC	https://www.proteinatlas.org/ENSG00000186111-PIP5K1C/pathology/thyroid+cancer
	Normal	https://www.proteinatlas.org/ENSG00000186111-PIP5K1C/tissue/thyroid+gland
HNRNPC	PTC	https://www.proteinatlas.org/ENSG00000092199-HNRNPC/pathology/thyroid+cancer
	Normal	https://www.proteinatlas.org/ENSG00000092199-HNRNPC/tissue/thyroid+gland
NUP54	PTC	https://www.proteinatlas.org/ENSG00000138750-NUP54/pathology/thyroid+cancer
	Normal	https://www.proteinatlas.org/ENSG00000138750-NUP54/tissue/thyroid+gland
NUP93	PTC	https://www.proteinatlas.org/ENSG00000102900-NUP93/pathology/thyroid+cancer
	Normal	https://www.proteinatlas.org/ENSG00000102900-NUP93/tissue/thyroid+gland
GEMIN5	PTC	https://www.proteinatlas.org/ENSG00000082516-GEMIN5/pathology/thyroid+cancer
	Normal	https://www.proteinatlas.org/ENSG00000082516-GEMIN5/tissue/thyroid+gland
FIP1L1	PTC	https://www.proteinatlas.org/ENSG00000145216-FIP1L1/pathology/thyroid+cancer
	Normal	https://www.proteinatlas.org/ENSG00000145216-FIP1L1/tissue/thyroid+gland
SNRPD3	PTC	https://www.proteinatlas.org/ENSG00000100028-SNRPD3/pathology/thyroid+cancer
	Normal	https://www.proteinatlas.org/ENSG00000100028-SNRPD3/tissue/thyroid+gland
NUPL1	PTC	https://www.proteinatlas.org/ENSG00000139496-NUP58/pathology/thyroid+cancer
	Normal	https://www.proteinatlas.org/ENSG00000139496-NUP58/tissue/thyroid+gland

Abbreviation: PTC, papillary thyroid carcinoma.
